# A Framework for Determining the Performance and Requirements of Cable-Driven Mobile Lower Limb Rehabilitation Exoskeletons

**DOI:** 10.3389/fbioe.2022.920462

**Published:** 2022-06-20

**Authors:** Rajan Prasad, Marwan El-Rich, Mohammad I. Awad, Irfan Hussain, H.F. Jelinek, Umer Huzaifa, Kinda Khalaf

**Affiliations:** ^1^ Department of Mechanical Engineering, Khalifa University of Science Technology and Research, Abu Dhabi, United Arab Emirates; ^2^ Health Engineering Innovation Center, Khalifa University of Science Technology and Research, Abu Dhabi, United Arab Emirates; ^3^ Khalifa University Center for Autonomous Robotic Systems (KUCARS), Khalifa University of Science Technology and Research, Abu Dhabi, United Arab Emirates; ^4^ Department of Biomedical Engineering, Khalifa University of Science Technology and Research, Abu Dhabi, United Arab Emirates; ^5^ Center for Biotechnology, Khalifa University of Science Technology and Research, Abu Dhabi, United Arab Emirates; ^6^ School of Computing, DePaul University, Chicago, IL, United States

**Keywords:** lower limb rehabilitation, cable-driven exoskeleton, performance analysis, generalized framework, link-based model, tracking

## Abstract

The global increase in the number of stroke patients and limited accessibility to rehabilitation has promoted an increase in the design and development of mobile exoskeletons. Robot-assisted mobile rehabilitation is rapidly emerging as a viable tool as it could provide intensive repetitive movement training and timely standardized delivery of therapy as compared to conventional manual therapy. However, the majority of existing lower limb exoskeletons continue to be heavy and induce unnecessary inertia and inertial vibration on the limb. Cable-driven exoskeletons can overcome these issues with the provision of remote actuation. However, the number of cables and routing can be selected in various ways posing a challenge to designers regarding the optimal design configuration. In this work, a simulation-based generalized framework for modelling and assessment of cable-driven mobile exoskeleton is proposed. The framework can be implemented to identify a ‘suitable’ configuration from several potential ones or to identify the optimal routing parameters for a given configuration. For a proof of concept, four conceptual configurations of cable-driven exoskeletons (one with a spring) were developed in a manner where both positive and negative moments could be generated for each joint (antagonistic configuration). The models were analyzed using the proposed framework and a decision metric table has been developed based on the models’ performance and requirements. The weight of the metrics can be adjusted depending on the preferences and specified constraints. The maximum score is assigned to the configuration with minimum requirement or error, maximum performance, and *vice versa*. The metric table indicated that the 4-cable configuration is a promising design option for a lower limb rehabilitation exoskeleton based on tracking performance, model requirements, and component forces exerted on the limb.

## Introduction

According to World Health Organization (WHO), stroke is the second leading cause of mortality and the third leading cause of long-term disability worldwide (Johnson et al., 2016). In 2020, 13.7 million people around the world had their first stroke, and five and a half million consequently lost their lives (Learn about stroke, 2022). In general, up to 74% of stroke survivors have physical, cognitive, and emotional challenges leading them to become dependent in activities of daily living (ADL). Movement impairment post-stroke most often affects the upper or lower limb on one side of the body resulting in hemiparetic gait (Sheffler and Chae, 2015) which induces compensatory motion on the healthy, contralateral limb (Balaban and Tok, 2014). In hemiparetic gait, various spatiotemporal parameters, including velocity, step and stride length, stance on the paretic limb, and cadence are significantly compromised (Sheffler and Chae, 2015). This alteration primarily stems from impairment in motor control, muscle spasticity, and degraded strength, as well as abnormal muscle synergistic activation and interaction (Li et al., 2018). Moreover, the duration of the double support phase (DSP) and that of the paretic limb swing phase increase to maintain stability during walking. These changes in kinematic and spatiotemporal parameters often lead to asymmetric gait (Figueiredo et al., 2015) and result in further difficulties to complete ADL.

Rehabilitation improves ADL of stroke survivors and helps them walk independently by primarily focusing on regaining optimal muscle and nervous system function towards functional gait restoration. Stroke rehabilitation should start as early as possible. It should be repetitive and intensive, task-oriented, proportional to the patient’s functional status, and designed to encourage and motivate the patient with the help of feedback (Winstein et al., 2016). Due to its multiple potential advantages over manual therapy, including automated, accurate, repetitive intervention, and the potential for tele and home rehabilitation solutions, robotic rehabilitation has recently been at the forefront of stroke rehabilitation research (Unluhisarcikli et al., 2011; Chen et al., 2013; Riener, 2016). Robots not only could provide task-based high intensity, repetitive functional movement training, but could also offer continuous and objective quantitative movement assessment, as well as economical solutions for large-scale use (Dao and Yamamoto, 2018). The last decade has witnessed an increasing focus on exoskeletons and rehabilitation devices. A remarkable number of powered and portable (autonomous power supply) lower-limb exoskeletons were designed to serve as assistive devices for ADL attainment as well as gait rehabilitation. The majority of these rehabilitation devices could be categorized as a tethered power supply (stationary (treadmill-based, or footplate-based); moving (over ground-based)); and portable (autonomous power supply). Most devices function by actuating joints directly (i.e., LOKOMAT (Riener, 2016), ANdROS (Unluhisarcikli et al., 2011), AIRGAIT (Dao and Yamamoto, 2018), and AGoRA (Sanchez-Manchola et al., 2018), etc.) where the actuators are mounted *via* links near the joints and the actuator along with the link induces both inertia and inertial vibration to the attached stroke-affected limb. Furthermore, direct actuation at the joint level requires strong structural support on the acting and reacting parts, which renders these systems heavy and bulky. In such designs, the system also tends to be less transparent as it compensates for the user as well as for the component (support and actuators) weights. In the direct actuation approach, the knee joint is assumed as a pin joint, which oversimplifies its biomechanics and may induce unnecessary moments (Wang et al., 2014) at the joint. Importantly, safety issues need to be taken into consideration when placing an actuator directly at a physiological joint, as the device closely interacts with the human body and any misalignment may induce undesired moments or torques, which could compromise the safety and comfort of the user.

Lower limb exoskeletons with remote actuator locations (distant from the joint), not only enhance the human interaction safety and function of the exoskeleton but also reduce the inertial vibration at the joint (Grosu et al., 2018). However, reducing or compensating alone for the exoskeleton’s structural weight is not sufficient for restoring natural gait due to dominant inertial vibrations (Jin et al., 2017). Thus, to restore natural gait more closely, both weight and inertia-induced vibrations need to be minimized. Cable-driven rehabilitation devices (CDRD) were introduced to overcome these limitations (Bryson and Agrawal, 2014; Jin et al., 2015; Alamdari and Krovi, 2016; Witte et al., 2017). For Example, employing Bowden cables as transmission elements facilitate the remote location of the actuators and minimize the inertia and inertial vibration on the impaired limb as well as the need for strong structural support. Actuating *via* cables acts in a similar manner to an end effector (actuated by exerting force/moments at the end) and hence mitigates the burden of exact joint alignment.

C-ALEX (Jin et al., 2015) is an example of a 4 cable-driven rehabilitation device in which cables are routed through multiple cuffs mounted on the lower limb. An optimization-based strategy distributes the cable tensions to ensure that they are always taut, within the specified limit, and meet the required joint torque requirement at the joint. The robot employs both PD (proportional-derivative) and force-field controller to track the user’s ankle motion during training. Experiments on healthy subjects revealed that C-ALEX assisted users to track the desired motion of the limb with the selected ankle trajectory. Kirby et al. (Witte et al., 2017) designed and tested a cable-driven knee rehabilitation exoskeleton using a powerful tethered testbed. The rehabilitator had a lightweight structure (0.76 kg) achieved by mounting the actuators and control accessories on a testbed which transmitted power to the rehabilitator *via* two cables allowing for rapid design modification in design. Although the device showed its potential as a knee rehabilitator, rehabilitating the knee joint alone was sufficient to regain a healthy gait trajectory in stroke patients. Bryson et al. (Bryson and Agrawal, 2014) analyzed a 3 DOFs robot leg actuated device using 4 cables to identify the optimal parameters most commonly associated with performance, such as cable routing and configuration. The configuration fulfilling the design requirements based on the analysis was used in designing the cable-driven robot. Aliakbar et al. (Alamdari and Krovi, 2016) designed a 4 cable-driven lower limb rehabilitation exoskeleton which tracked the lower limb joints and positions, employing a (PD) controller and force field controller respectively. The cables were routed from the fixed frame (attached to the treadmill) to the lower limb, where one cable was assigned for the hip and knee joint while the remaining cables were assigned for ankle tracking. The design constrained the overall device mobility along with the routing and configurability of cables.

Although it is well accepted that CDRD has immense potential in robotic rehabilitation, the optimal cable routing(s) and configuration toward enhancing design options and human safe interaction and comfort remain elusive. In the literature, cable configurations which meet the desired performance specifications and objectives while minimizing the system requirements have not been fully achieved, and there continues to be a lack of generalized methodology of modelling to accommodate a variety of configurations and routings. In this work, we purpose a generalized methodology/framework to evaluate the feasibility of various routings and configuration-based designs of cable-driven exoskeletons and provide metrics to help in selecting the suitable configuration for an optimal design based on the specified constraints.

## Modelling of Cable Driven Exoskeletons

**FIGURE 1:**
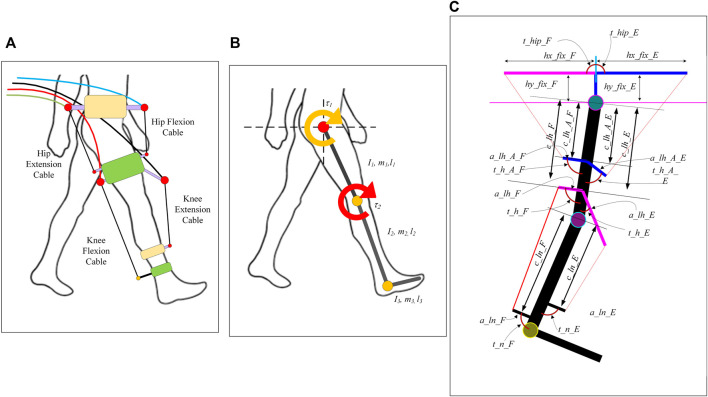
Conceptual cable driven model **(A)**, 2 link-based model of lower limb **(B)**, and transformation of conceptual cable driven exoskeleton model into the link-based model **(C)**.

In our previous work (Prasad et al., 2022), we proposed a generalized framework for modelling a cable-driven exoskeleton. The proposed model can test the feasibility and performance of a variety of configurations and routing of cables. However, the framework assumed the hip cuff to be oriented at a fixed angle to the pelvis. In the current study, the framework has been extended to accommodate the hip cuff at any angle. This adaptation makes the model more realistic, modular, and configurable. Furthermore, a three-variable definition-based strategy was selected to fully define any cuff on the user’s body. The framework assumes the lower limb as a 2-link model (thigh and shank) with the foot being fixed perpendicular to the shank (Figure 1). Exoskeleton assistance is provided only during the swing phase of the motion assuming that the person can continue the stance phase on their own. For the sake of simulation, the leg is assumed to be suspended in air replicating the swing phase.

The equation of motion formulated in MATLAB using Euler- Lagrange can be written as:

$$M\left(\theta \right)\ddot{\theta }+C\left(\theta ,\dot{\theta }\right)\dot{\theta }+G\left(\theta \right)=\tau$$ (1)

Where *M(θ)* represents the inertial matrix (2 × 2), $C\left(\theta ,\dot{\theta }\right)$ represents the Coriolis component (2 × 1), *G(θ)* represents the Gravitational components (2 × 1), and τ represents the torques on the joints.

### Generalized Cuff Parameters

The model was created such that any cuff placed on the user’s limb (either pelvis, thigh, or shank) can be defined using three parameters as shown in Figure 1C. ***F*** and ***E*** represent the posterior (back) and anterior (front) sides of the leg. The measured angles are assumed positive in the clockwise direction.

The three defined parameters are

1) The distance of the cuff from the nearest joint (hip for thigh and pelvis, knee for shank cuffs).
2) The length of the cuff.
3) The angle of the cuff from the attached limb part (measured by viewing from the cuff to the nearest joint).

For example, the shank cuff on the posterior side can be fully defined using three parameters *c_ln_F, a_ln_F, and t_n_F* respectively. All the other cuffs can be defined similarly.

### Control System

**FIGURE 2:**
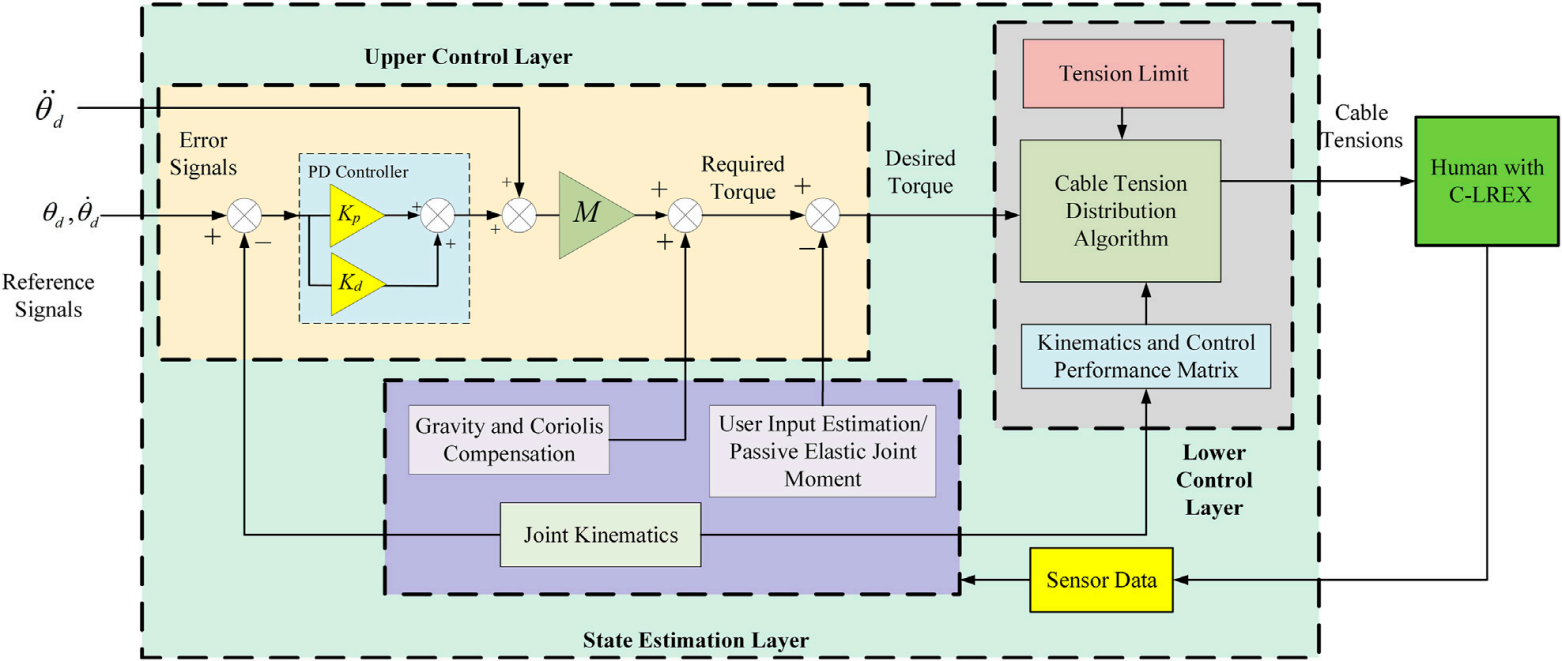
Generalized control strategy.

The controller used in this study is an impedance controller where a PD controller is employed to estimate the desired torque based on the errors, similar to controllers used in orthoses (impedance control) reported in (Unluhisarcikli et al., 2011) (Riener et al., 2005) (Tucker et al., 2015). The desired trajectory during motion is tracked using a three-layer control algorithm based on the 2-link model. Figure 2 depicts the generalized control strategy with the all-control layers. C-LREX stands for the Cable driven Lower limb Rehabilitation Exoskeleton.

#### Upper Control Layer

The upper control layer is responsible for generating the desired torque required for tracking the next step based on the current motion step. It can employ various controllers such as PID (proportional—integral-derivative), MPC (model predictive control), SMC (sliding mode control), etc. In the current controller, PD-based feedback linearization was used to transform the error dynamics system into an exponentially stable system and is defined as: $\tau =M\left(K_{p}e+K_{d}\dot{e}+{\ddot{\theta }}_{d}\right)+C\dot{\theta }+G$ Where $K_{p}$ and $K_{d}$ are diagonal matrices $K_{p}=diag\left(K_{p1},K_{p2}\right),\text{​}\;K_{d}=diag\left(K_{d1},K_{d2}\right)$ With this feedback linearization, the error dynamics can be written as: $K_{p}e+K_{d}\dot{e}+\ddot{e}=0$ .

The above feedback linearization yields the required torque to track the trajectory. The passive elastic joint moment is considered as the user’s voluntary contribution during walking and is subtracted from the required torque to estimate the desired torque, which is then re-distributed among the cables *via* the cable tension distribution module.

#### State Estimation Layer

The state estimation layer is responsible for identifying current angles, angular velocities, inertial and other matrices (*M, C, G*), as well as the user’s voluntary input based on the sensor’s data. The sensor data may include kinematics, kinetics, or both depending on the requirements (angle, angular velocity, angular acceleration, and cable tension). The matrices are calculated using kinematics data and the 2-link model equations listed in Eq. 1. At present, the model considers passive elastic joint moment as the user’s voluntary input during the motion. The anthropometric data required for the model is obtained using Winter’s (Winter 2009) model based on the user’s height and weight.

The active joint moment accounts for the active muscle contribution while the passive joint moment includes the contribution of passive muscles as well as other passive structures, such as ligaments and joint capsules. The moment is exerted to the distal segment about the joint center from the proximal segment. In literature, a double exponential-based model has been widely adopted with joint kinematics to predict the passive elastic joint (Riener and Edrich, 1999, 2013; Amankwah et al., 2004; Whittington et al., 2008). Apart from the double exponential-based model, some other models, such as those combining linear and exponential-based functions, have also been proposed (Hines et al., 2018). The study conducted by (Gasparutto et al., 2018) with models proposed in the literature (Riener and Edrich, 1999; Amankwah et al., 2004) found that the passive elastic joint moment predicted by both models are somehow similar in pattern and alignment with the literature data, suggesting that these models can be used even though they are a non-subject-specific. In this study, the passive elastic joint moments were estimated based on the model in work (Riener and Edrich, 1999).

#### Lower Control Layer

The lower control layer interacts directly with the exoskeleton and is responsible for generating the desired control effect by distributing various tensions among the cables based on the capability and limitation of the system at that instant of motion.

##### Control Performance Matrix Estimation

The C-LREX employs cables to generate the required torque at the hip and/or knee joints and the relationship between the cable tension and the torque generated can be derived by either using the principle of virtual work (as derived in work (Jin et al., 2015)) or *via* vector algebra methods. Both methods yield the same output, however, the vector algebra approach provides shear and compressive forces that act at the joints while calculating the torques induced *via* the cables.

**FIGURE 3:**
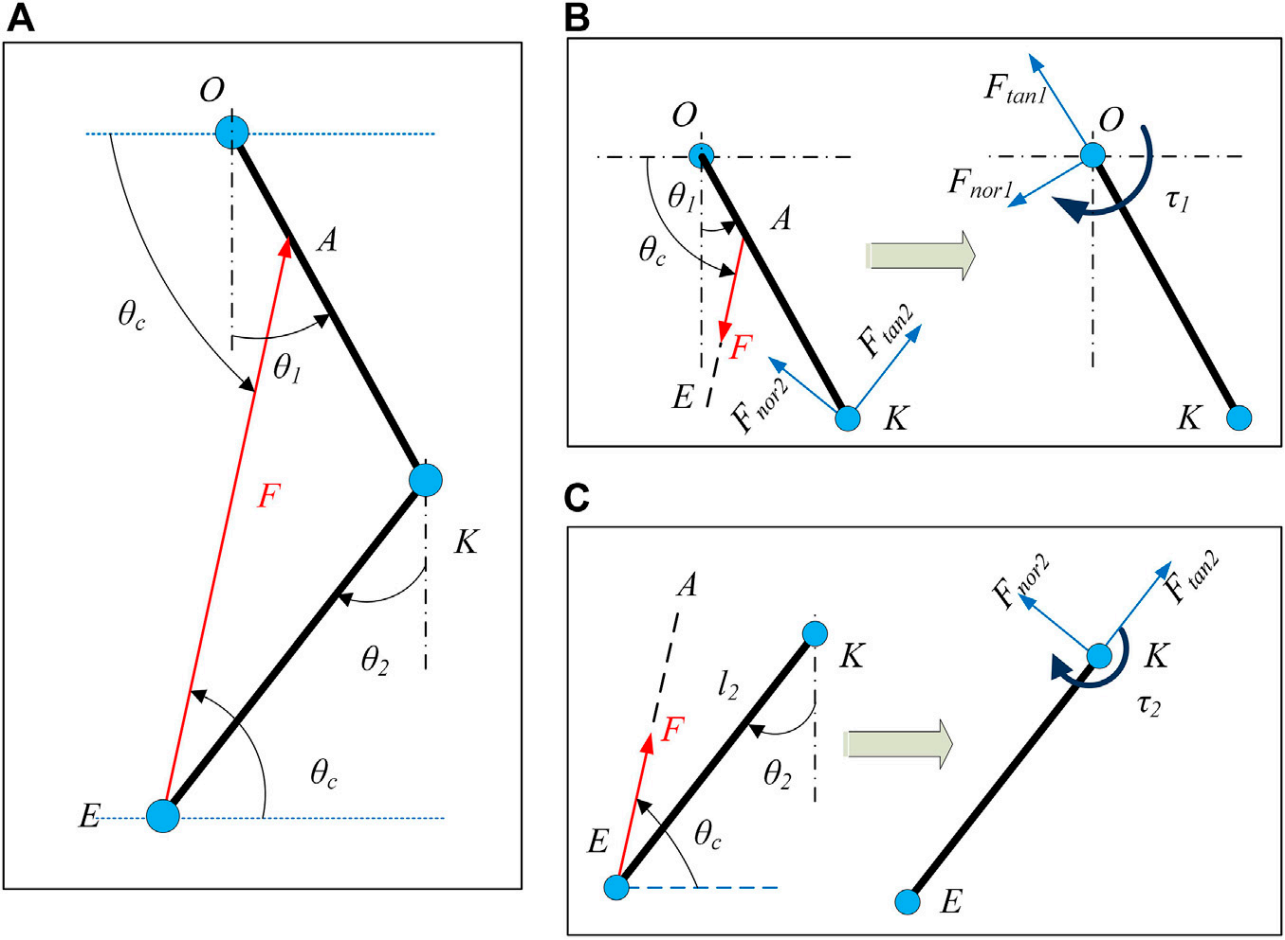
Two link model with applied cable tension **(A)**, Force to Torque mapping for shank **(C)**, and Force to Torque mapping for thigh **(B)**. *F*_*tan*_ and *F*_*nor*_ represent the compressive and shear forces respectively induced on the joints. Subscripts 1 and 2 represent the hip and knee joints respectively.

The cable tension vector is projected onto the shank and thigh vectors to find the tangential and normal component of the force as shown in Figure 3. The projection of the cable tension vector ( $\vec{EA}$ ) along the shank vector provides the tangential component of the force, while the orthogonal component of ( $\vec{EA}$ ) provides the normal force component. The torque on the joint that is caused by the cable tension can be estimated using the cross-product of the distance vector and the force vector.

$$\begin{array}{l} \vec{F_{\tan2}}=\left(proj_{\hat{EK}}\hat{EA}\right)=\left(\frac{\left(\hat{EA}•\hat{EK}\right)}{{\left\|\hat{EK}\right\|}^{2}}\hat{EK}\right)\\ \vec{F_{nor2}}=\left(\hat{EA}-proj_{\hat{EK}}\hat{EA}\right)=\left(\hat{EA}-\frac{\left(\hat{EA}•\hat{EK}\right)}{{\left\|\hat{EK}\right\|}^{2}}\hat{EK}\right)\\ \vec{\tau_{2}}=\left(\vec{KE}\times \hat{EA}\right) \end{array}$$ (2)

Where, $\hat{EA}\;and\;\hat{EK}$ are the unit vector along the $\vec{EA}\;\;and\;\vec{EK}$ direction, respectively.

Similarly, the equivalent force components and torque on the hip joint can be estimated as:

$$\begin{array}{l} \vec{F_{\tan1}}=\left(proj_{\hat{KO}}\vec{F_{\tan2}}\right)+\left(proj_{\hat{KO}}\vec{F_{nor2}}\right)+\left(proj_{\hat{KO}}\hat{AE}\right)\\ \vec{F_{nor1}}=\vec{F_{\tan2}}-\left(proj_{\hat{KO}}\vec{F_{\tan2}}\right)+\vec{F_{nor2}}-\left(proj_{\hat{KO}}\vec{F_{nor2}}\right)\\ \;\;\;\;\;\;\;\;\;\;\;\;\;\;\;+\hat{AE}-\left(proj_{\hat{KO}}\hat{AE}\right)\\ \vec{\tau_{1}}=\left(\vec{OK}\times \vec{F_{\tan2}}\right)+\left(\vec{OK}\times \vec{F_{nor2}}\right)+\left(\vec{OA}\times \hat{AE}\right) \end{array}$$ (3)

In Eqs 2, 3, the actual values of force and torque are obtained by multiplying the outcomes of the equations with the magnitude of the applied cable tension. In Eqs 2, 3, the force magnitude is unknown for a given cable routing configuration.

Thus, the relation between torque and force can be written as:

$$\tau =\left[\begin{array}{c} \tau_{1}\\ \tau_{2} \end{array}\right]=\left[\begin{array}{c} B_{1}\\ B_{2} \end{array}\right]F=B^{T}F$$ (4)

Where, $B^{T}={\left[\begin{array}{cc} B_{1} & B_{2} \end{array}\right]}^{T}$ is the control performance matrix that maps the cable tension *F* to the hip joint and knee joint torque and is dependent on the kinematics of the C-LREX at any instant.

In general, C-LREX employs a combination of cables to generate the desired control effect. The dimension of matrix ***B*** depends on the number of the cables. For example, using 3 separate cables, the control performance matrix (***B***) can be written from Eq. 4 as:

$$F={\left[\begin{array}{ccc} F_{1} & F_{2} & F_{3} \end{array}\right]}^{T},\tau ={\left[\begin{array}{cc} \tau_{1} & \tau_{2} \end{array}\right]}^{T}$$

$$B={\left[\begin{array}{ccc} J_{1} & J_{2} & J_{3} \end{array}\right]}^{T}={\left[\begin{array}{ccc} J_{11} & J_{21} & J_{31}\\ J_{12} & J_{22} & J_{32} \end{array}\right]}^{T},\text{​}\;B\in {\mathbb{R}}^{3\times 2},J\in {\mathbb{R}}^{2\times 1}$$

##### Cable Tension Distribution Algorithm

The cable tension can be distributed either in a priority-based order or in an optimized order. The priority-based algorithm gives full priority to knee control allocation and then to hip control allocation in a single operational step. While the optimization-based algorithm distributes overall control based on a defined objective function in an iterative approach. Supplementary Figure S1 layout the cable tension distribution algorithm based on the priority approach and optimization.

The number of inputs and outputs for the control distribution is not always the same (depends on the number of cables) and results in a rectangular control performance matrix. Depending on the number of cables, the system could be either overdrive (if the number of outputs is greater than the input) or underdrive (if the number of outputs is less than the number of inputs). The tension in each cable for a given torque requirement must be coordinated in such a way that either the cable generates the exact torque or less than the required torque while utilizing the full potential features of the configuration and actuators. Since the control performance matrix (***B***) is not square, the values of the corresponding tensions in the cable cannot be determined easily as the inverse of ***B*** does not exist. Thus, it is essential to form an optimized control allocation strategy, although, optimization-based distribution requires additional resources. In the case where constraints are not tightly aligned, and faster distribution is required, a priority-based control allocation approach can be implemented.

The objective function for optimization can be defined in different ways. Eq. 4 can be transformed into a simple optimization problem as:

$$\left\{ \begin{array}{l} \tau =BF\\ F_{\min}\leq F\leq F_{\max} \end{array}\right.$$ (5)

Where *F*_min_ *and F*_max_ are the lower and upper bounds for the values of cable tension(s).

To solve the above problem, MATLAB-based *find minimum of constrained nonlinear multivariable function (fmincon)* function can be implemented. The objective function is defined as the minimization of allocated control to the actuators as:

$$\begin{array}{l} \underset{F}{\min}\left(FF^{T}\right)\\ s.t.\;\left\{ \begin{array}{l} \tau =BF\\ F_{\min}\leq F\leq F_{\max} \end{array}\right. \end{array}$$ (6)

Based on the previous work reported in the literature (refer to (Prasad et al., 2020) for detailed formulation and solution), the simple optimization problem (Eq. 6) can be converted into a quadratic programming (QP) problem. QP objective function is defined as a hybrid objective function (combination of error minimization and control minimization) and is transformed to have a final form as a standard QP function in Eq. 7. The solution is obtained either by employing MATLAB QP solver or the algorithm described (Prasad et al., 2020).

$$\begin{array}{l} \underset{F}{\min}\left(\frac{1}{2}F^{T}HF+f^{T}F\right)\\ s.t.\;\{AF\leq D \end{array}$$ (7)

Where $A={\left[\begin{array}{cc} -I & I \end{array}\right]}^{T},\;D={\left[\begin{array}{cc} -F_{\min} & F_{\max} \end{array}\right]}^{T}$ , ***H*** is the Hessian matrix and ***f*** is the gradient matrix calculated from the hybrid objective function.

## Conceptual Models

The modelling approach described in the previous section outlines a generalized framework that can be exploited to assess the viability of CDRD, identify the model requirements, and compare their performances. This section focuses on the implementation of the generalized framework to develop a decision metric that can be used to select a suitable cable routing configuration among a group of alternative configurations. Throughout the literature, a 4-cable configuration has been most often adopted for a mobile cable-driven rehabilitation device. However, other configurations can also meet similar performance requirements. The number of cable configurations that can be generated remains therefore elusive and depends on multiple factors such as design requirements, power constraints, performance requirements, and so on.

### Selection of the Conceptual Models

**FIGURE 4:**
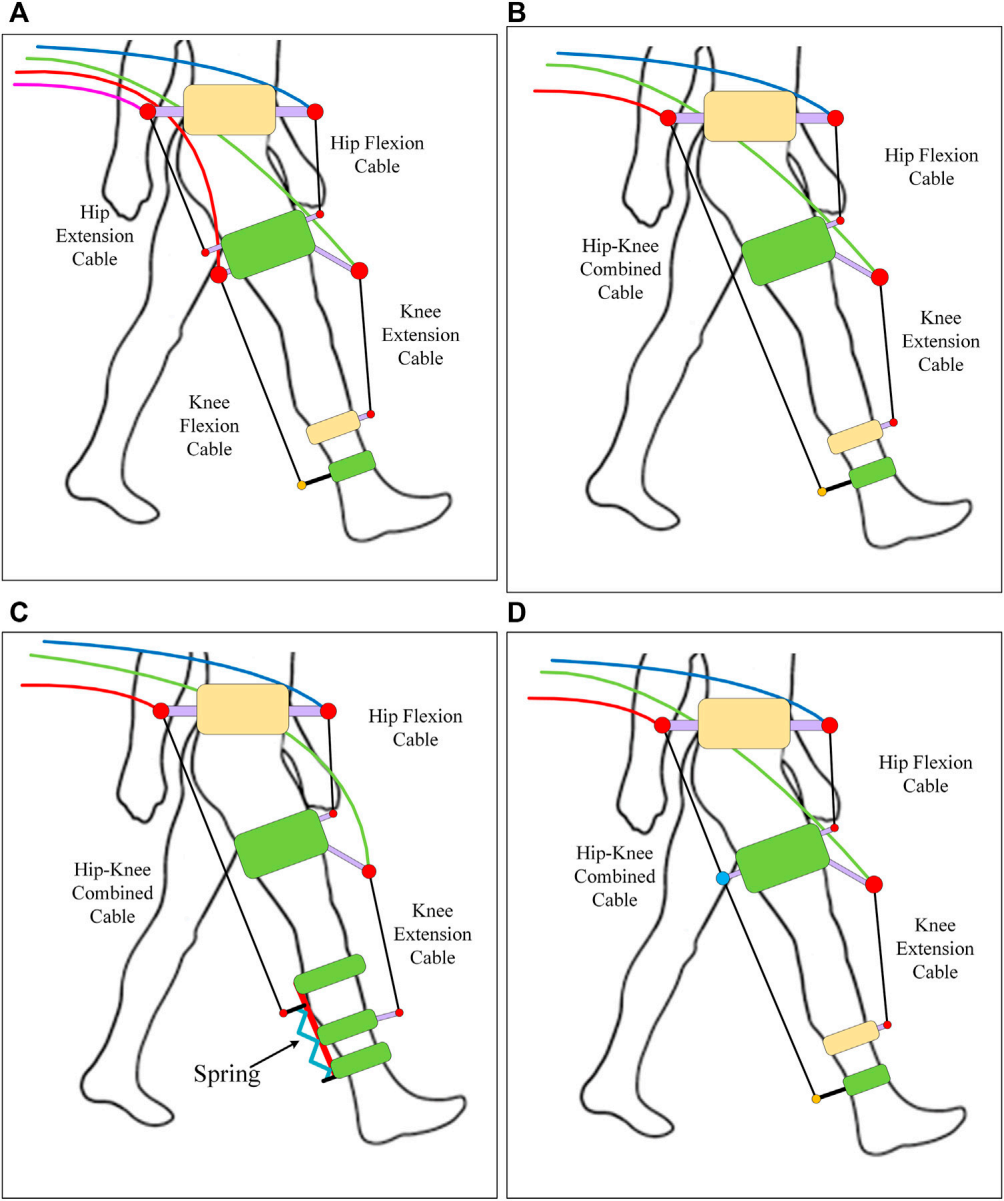
Conceptual models: **(A)** Configuration 1 **(B)** Configuration 2, **(C)** Configuration 3, and **(D)** Configuration 4.

The optimal exoskeleton/rehabilitation device applies the required torques on the hip and knee joints and satisfies the required coordination between the motion of the limb and joints to achieve the desired gait patterns. Hence, to track the desired motion, the cables need to be configured in a manner such that each joint can generate the required joint moment (either independently or in relation with other joints) in both flexion and extension directions (if only sagittal motion is considered). Failure to provide the moment in either direction at either joint (the hip or knee joint) will result in poor coordination and thus lead to a higher deviation from the desired trajectory ((Prasad et al., 2022)). To accomplish this, the cables must be arranged in an antagonistic configuration where 2 cables (either individually or combined) act at a joint in opposite directions to provide flexion and extension torque. Four examples of conceptual models capable of generating torque on joints in antagonistic configurations are selected (Figure 4) for demonstration in this study. Since springs are passive energy storing elements which can alter the energy cycle of a system, a spring was added to the 3-cable model to study its effect on the performance and requirements of the model (Figure 4C).

Configuration 1: 4 cables where 2 cable acts for each joint (hip and knee) separately in antagonistic configuration. Configuration 2: 3 cables where the posterior cables of configuration are combined as one acting from pelvis to shank. Configuration 3: an extension of configuration 2 where spring is attached to the shank cuff that allows movement along the shank only depending on the magnitude of applied cable tension. Configuration 4: 3 cables where the combined Hip-Knee cable is routed through the thigh cuff and thus acts as two cables with the same cable tension.

### Modelling of the Spring

A second-order spring with stiffness and damping coefficients is used in the model (Eq. 8). The spring is attached to the posterior shank cuff from the foot side as shown in Figure 4C.

$$ms^{2}+bs+k=F$$ (8)

Where, ***m, b,*** *and* ***k*** represent mass, damping coefficient, and spring stiffness respectively. ***F*** is the force applied to the spring.

The spring is allowed to slide along the shank direction only in an upward/downward direction. The stiffness and damping constants are assumed as 500N/m and 10 N-s/m respectively. A dead-end (max stretchable length) of the spring is defined to simulate a physical stop or spring stop end.

### Simulating the Conceptual Models

The conceptual models (in Figure 4) are transformed into a link-based model with cuff parameters as shown in Figure 1. The link-based model is the same for all configurations (Figure 1B) while the number of cables only altered the number of cuffs (Figure 1C). The cuff parameters are kept the same for all configurations. The healthy reference trajectory is adopted from Fukuchi (Fukuchi et al., 2018) with a gait cycle time of 3.48 s of overground walking (plotted in Supplementary Figure S2). The maximum and minimum allowable cable tensions are set to 100N and 7N respectively, to ensure that the tension is within the desired range and the cable is always taut. Based on the assumptions and simplifications, a *MATLAB* model was developed and simulated to track the healthy gait trajectories (performances) and the requirements were analyzed. The models were simulated for 2 gait cycles.

## Decision Metrics Development

The conceptual model’s performance and the requirements during tracking are analyzed and developed as metrics to help in the decision-making process. Further information, such as the exertion of the component forces on the joints due to cable tension, is also included as additional metrics. Throughout the results, the solid line represents the swing phase, and the dashed line represents the stance phase of the gait cycle.

### Performance Analysis

Performance is one of the key metrics which plays a critical role in decision-making. Performance parameters, such as joint angle and angular velocity tracking, tracking error, root mean square error (RMSE), and ankle trajectory can be used to assess the performance of the models.

#### Joint Angle Error

**FIGURE 5:**
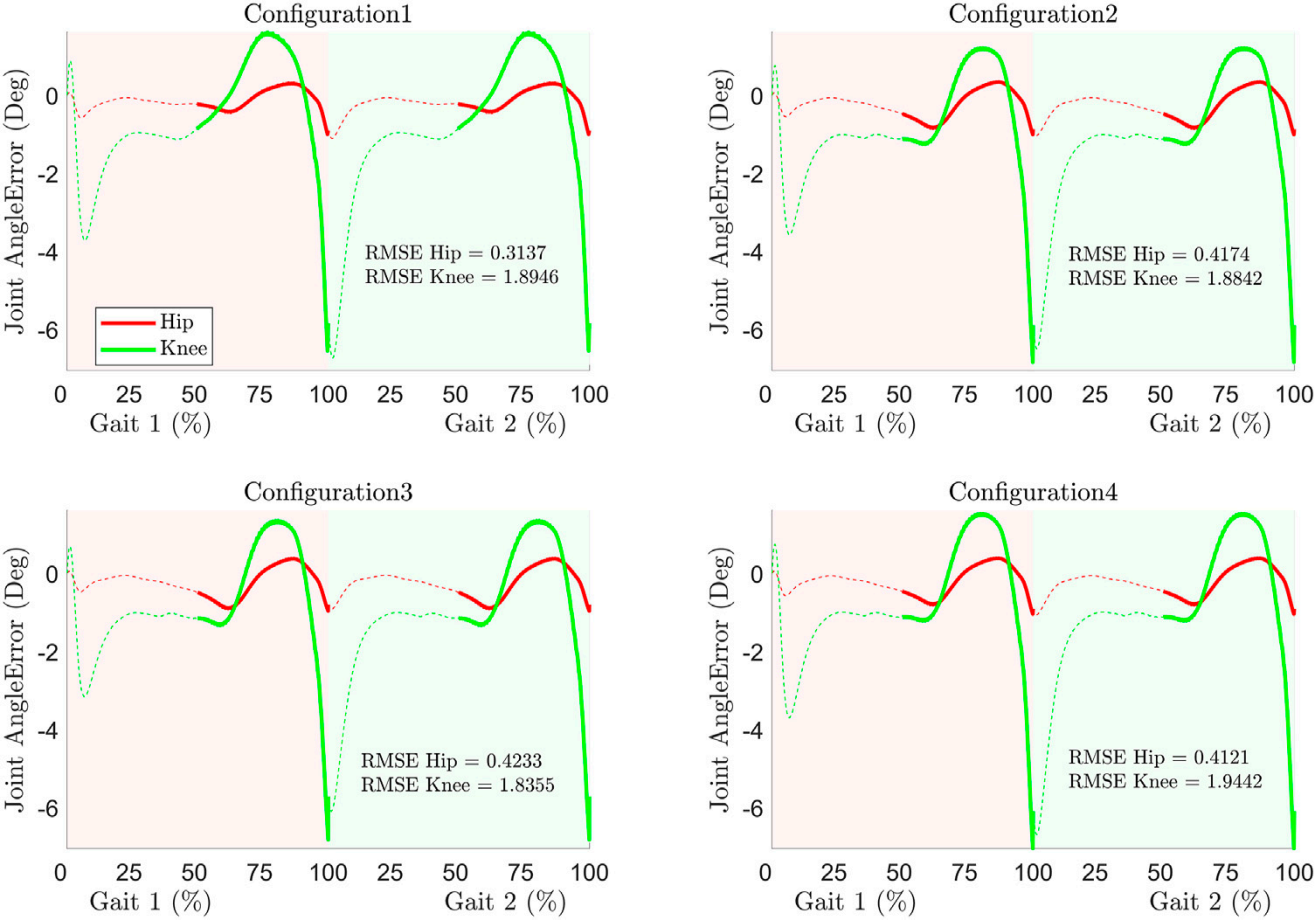
Joint angle error and RMSE during tracking.

The error in tracking the joint angle in all configurations and its RMSE (in degree) are shown in Figure 5. As depicted in the figure, the maximum error does not exceed 7° (in the knee joint at the transition between gait cycles (around 100% of the first gait cycle) while its counterpart in the hip joint is less than 1°. The tracking performance in each case is reasonable. The RMSE in the tracking for each model indicates that configuration 1 has better tracking than others for the hip joint while configuration 3 has better tracking for the knee joint.

#### Joint Angular Velocity Error

**FIGURE 6:**
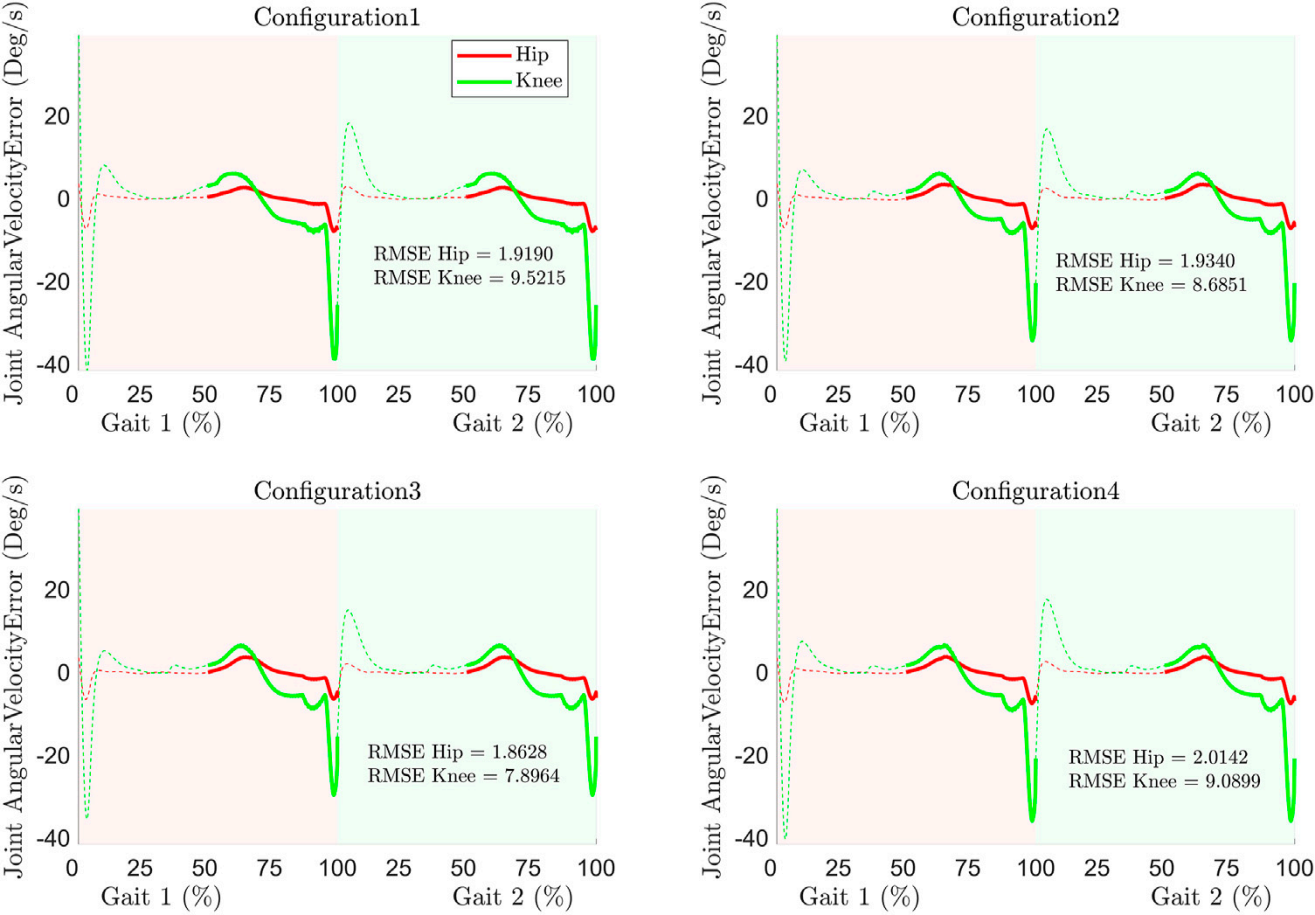
Joint angular velocities error and RMSE during tracking.

Similar to joint angle error, the joint angular velocity error during tracking (Figure 6) revealed a higher error in the knee joint in contrast to the hip joint. At the start of the gait cycle, the errors are higher due to the assumption of zero initial velocity. Furthermore, the errors are higher at the transition between the gait cycle (around 100% of the first gait cycle). The velocity requirement of the knee joint during the swing phase is higher and leads to higher inertial torque contribution. Since C-LREX has limited capability to exert torque (due to predefined cable tension range), the system fails to exert the required higher amount of torque and thus results in a higher error at the end of gait cycles. Nevertheless, the 3 cables configuration with spring demonstrated the least maximum velocity error. The RMSE (degree/s) of the joint velocity error (Figure 6) revealed that the 3 cables configuration outperforms others due to the spring contribution. The addition of a spring increased the flexibility of the attached cuff and contributed to additional velocity.

#### Ankle Position Tracking

**FIGURE 7:**
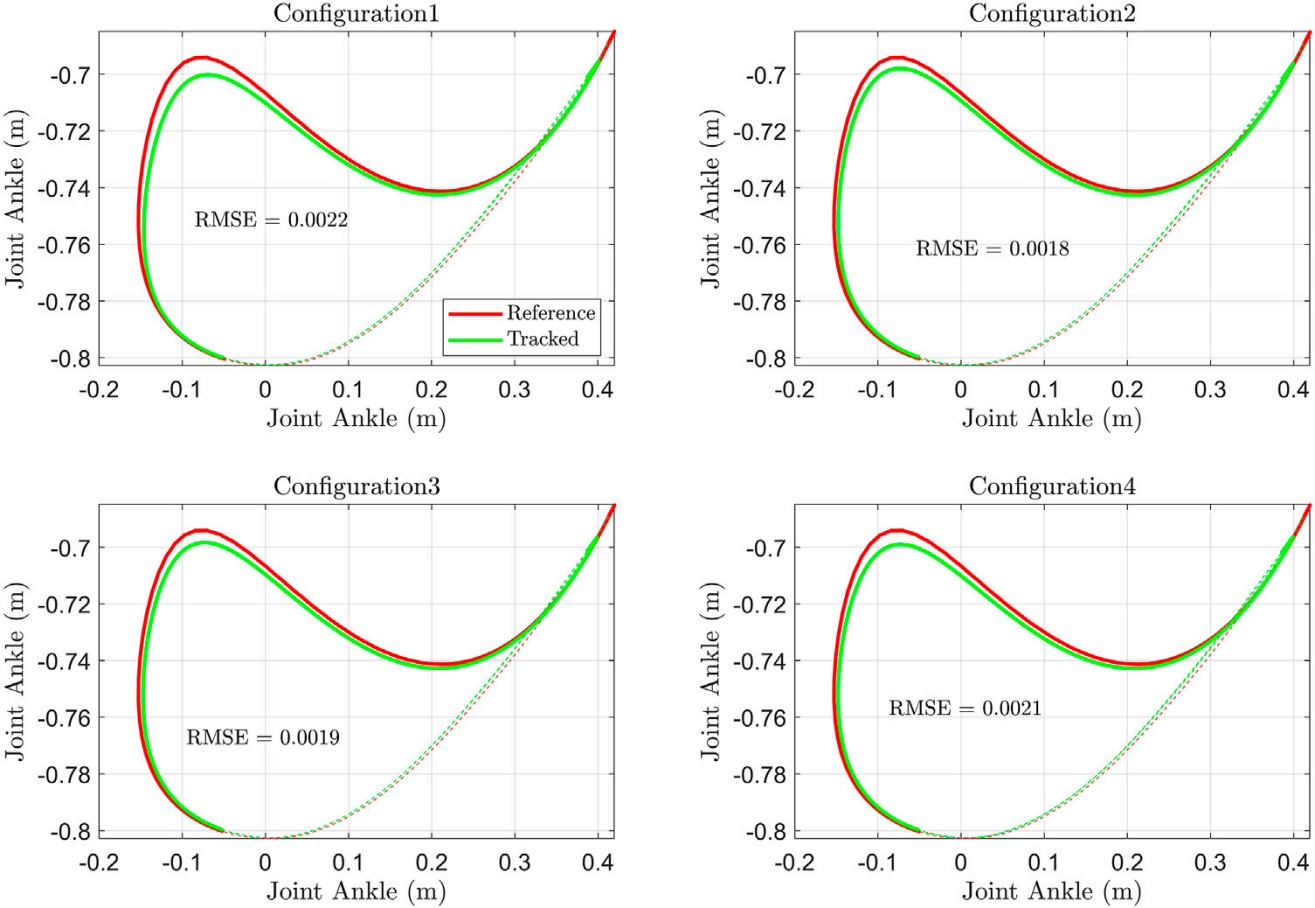
Ankle joint position (Hip joint as reference) and RMSE of the ankle distance tracking error.

The tracking of the ankle position (hip joint as origin) during tracking and the RMSE of the error of the distance of the ankle joint from the hip joint (hip as reference) are shown in Figure 7. The tracking in configurations 2 and 3 is superior to others. The 4-cable configuration has a higher error in tracking ankle position. The RMSE revealed that configuration 2 is tracking the ankle position more closely than other configurations.

### Requirement Analysis

The requirements to meet the above tracking performances in each configuration differ due to the different number of cables and routings. Requirements such as cable tension, motor torque, motor speed, motor power, and total power requirements can be analyzed to assess the configuration.

#### Cable Tension

**FIGURE 8:**
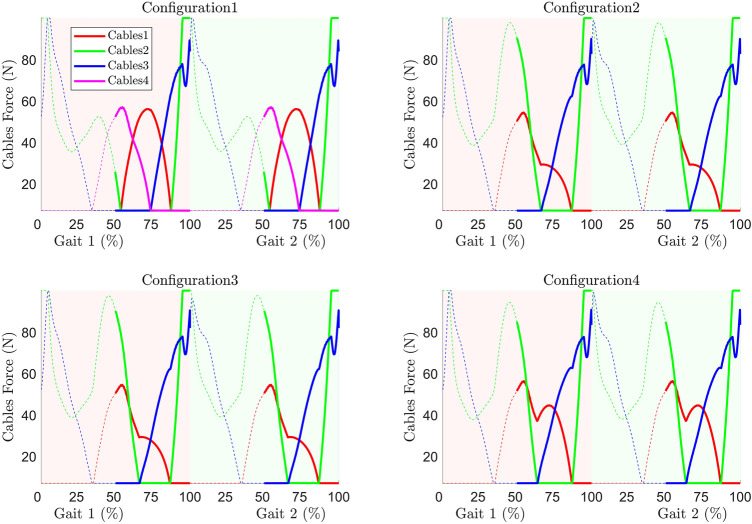
Cable tension requirement during trajectory tracking.

The cable tension(s) in each configuration is shown in Figure 8. The tension requirement in all configurations except for configuration 1 is higher in general throughout the gait cycle. Configurations 2 and 3 have a similar trend of cable tension distribution.

#### Cable Force Versus Cable Velocity Curve (Actuator Requirement Curve)

**FIGURE 9:**
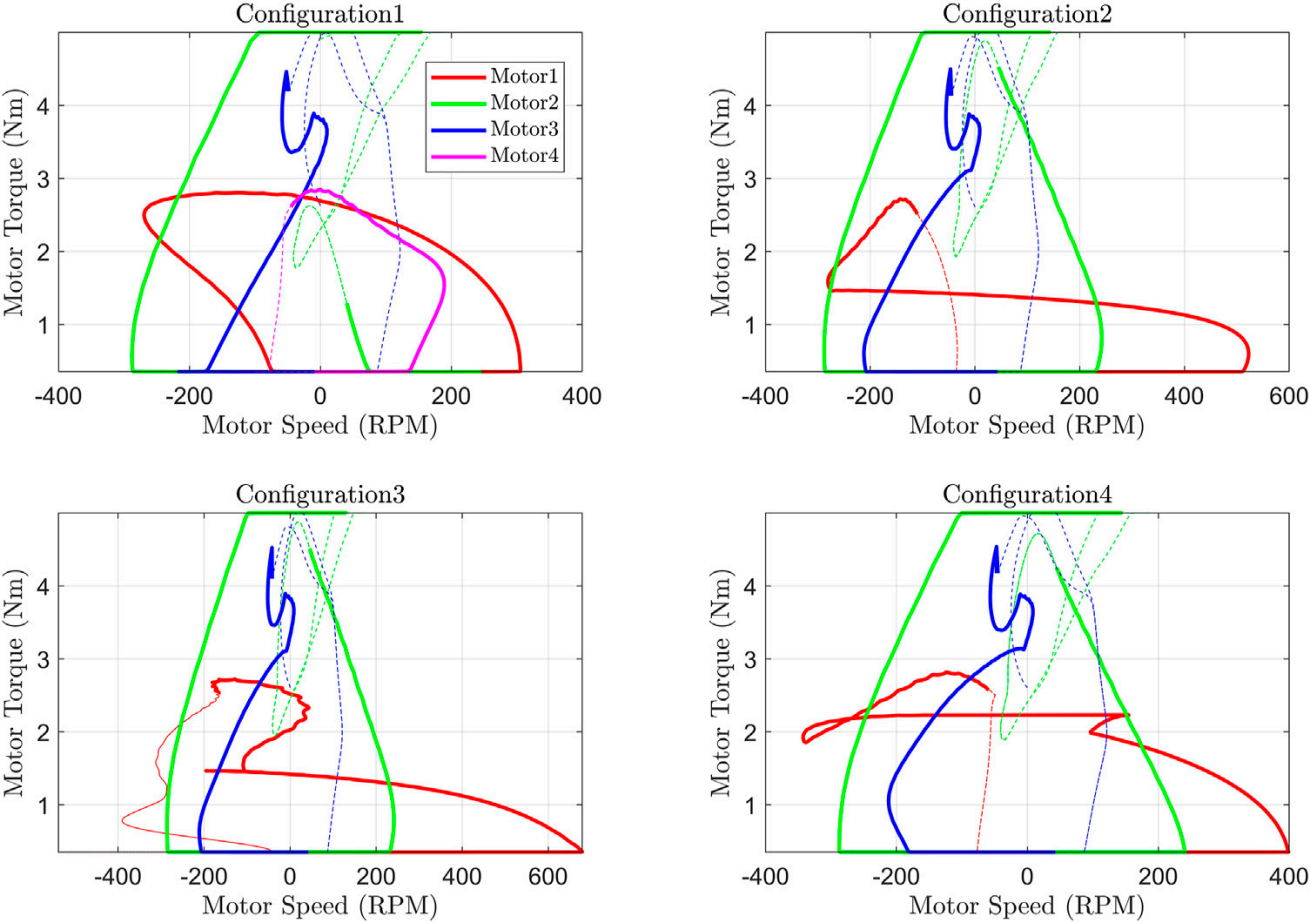
Motor torque versus speed curve.

The cable tension versus cable linear velocity (in Supplementary Figure S3) curve combines two requirements (tension and velocity) for a given cable of a configuration to meet the tracking performance. Since the cable tension is provided by the motor, this plot is important in the identification of actuators/motors. The cable tension can be converted into motor torque assuming a suitable cable roller diameter (assumed as a 5 cm radius in this study) attached to the motor. Similarly, the cable’s linear velocity can be transformed into motor speed (RPM) *via* the same roller diameter. The plots in Supplementary Figure S3 can be transformed into Torque-vs-Speed curves of the motor (actuator requirement curve) as shown in Figure 9. The area under these curves yields the total power requirement of each motor and the sum of the area under the curve is the total power required of the respective configuration, as listed in Table 1. The maximum torque requirement in each configuration is the same (due to the fixed max tension limit), but the speed requirement is different. Configuration 1 has the least speed requirement of the motor while configuration 3 has the highest (due to the spring contribution). The higher speed requirement results in a higher power requirement of the motor and thus a bigger size of the motor.

**TABLE 1:** The area under Torque-Speed Curve (Total Power Requirement of each motor).

Config/Motor	Motor1	Motor2	Motor3	Motor4	Sum
Config 1	30.6	55.5	16.7	21.8	124.6
Config 2	9.5	26.3	76.1	—	111.9
Config 3	38.6	26.5	72.0	—	137.0
Config 4	18.0	72.7	28.0	—	118.8

#### C-LREX Power Requirements

**FIGURE 10:**
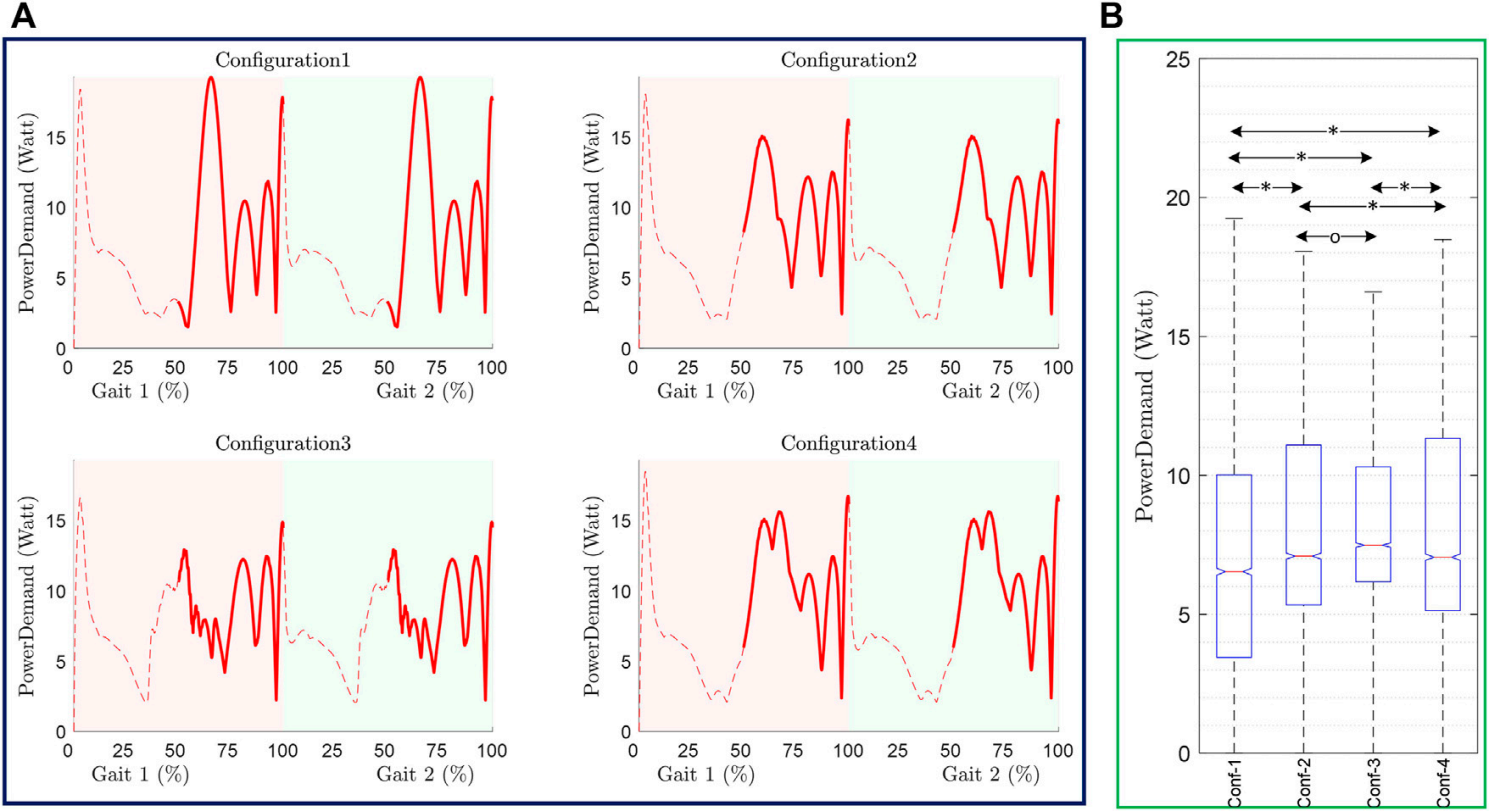
Power demand in C-LREX throughout Gait cycle **(A)** and one-way ANOVA analysis **(B)**. The pairwise significant difference and no significance between the configurations are marked as ‘*’, and ‘o’ respectively. *p-*value less than 0.05 is taken as a significant difference.

The number of cables corresponds to the number of motors in a configuration. The power requirements of the motor of each configuration (Supplementary Figure S4) are limited to 16 W; however, the patterns, as well as the number of motors in some configurations, are different. Since the number of the motors included in the exoskeleton will increase/decrease the power demand during the gait cycle, the sum of the individual motor’s power requirement is estimated to analyze the power demand pattern of the C-LREX and is shown in Figure 10 along with its statistical analysis (one-way ANOVA). The *p-value* in Figure 10B is less than 0.05 indicating that data are significantly different from each other. It was observed that configuration 3 has the highest total power requirement (Table 1) at least peak power demand (Figure 10). Configuration 1 has the highest peak power demand with a higher standard deviation (Table 2), while the mean peak power demand is the least among all configurations. Employing the spring has reduced the peak power demand and standard deviation, however with a higher median power demand in contrast to the configuration without a spring with 3 cables.

**TABLE 2:** Statistical analysis of Power demand of C-LREX in each configuration.

	Configuration	Mean ± Std	ANOVA	
			F	*p*
Power Demand	Config 1	7.44 ± 4.65	59.83	0.00
	Config 2	7.97 ± 3.83		
	Config 3	8.03 ± 2.95		
	Config 4	8.32 ± 4.12		

### Additional Metrics

Apart from the performance and the model requirements, it is also important to analyse and minimize the force exerted by the system on the user limb joints to ensure human-robot interaction, comfort, and safety. In general, a configuration should exert the least possible additional forces to meet the performance specification.

#### Joint Component Forces

**FIGURE 11:**
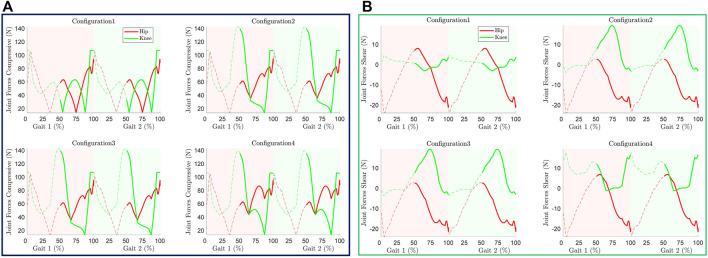
Joint component forces induced due to cable tension **(A)** Compressive, **(B)** Shear.

In this section, the joint compressive and shear forces resulting from the cable tensions were studied. These forces depend on the cable tensions and joint kinematics, and they vary with the gait cycle as shown in Figure 11. Configuration 1 exerts the least shear and compressive force on the knee joint compared to all other configurations while almost similar shear forces occur at the hip joint. Configuration 2 and 3 yields similar compressive and shear forces on the joints due to their similar cable tensions. The spring facilitates the movement of the posterior shank cuff in configuration 3 in contrast to configuration 2, however, it doesn’t alter the cable angle significantly and thus results in similar cable tension as well as component forces.

### Selecting a “Suitable” Configuration

Based on the metrics above and the design constraints, a suitable candidate from the four configurations studied here can be identified. For instance, in terms of performance (i.e., angle, angular velocity, and ankle position tracking), all the configurations are acceptable. On other hand, whilst considering the cable tension distribution throughout the cycle, configuration 1 is the “best”, however, it requires 4 motors as compared to the other configurations where 3 motors meet the demands. Furthermore, in terms of motor requirements, configuration 1 requires 4 motors with less power (thus smaller size) compared to others with 3 motors of higher power specification (thus bigger size). The higher power requirement is due to higher speed demand since the maximum torque is the same for all the configurations.

Similarly, for the total power required for the different configurations, configuration 2 is the ‘best’. The inclusion of spring has increased the power requirement by 22.43% while configuration 4 holds a reasonable position with a 6.16% higher requirement compared to configuration 2 (Table 1). In terms of individual motor power requirements, all configurations require less than 16 W. However, for peak power demand at a given instant of the motion, configuration 1 demands the highest peak power while other configurations without the spring remain close to configuration 1. The spring in configuration 3 results in the lowest peak power demand despite the maximum total power required throughout the gait cycle.

In terms of force exerted by the device on the lower limb joints, configuration 1 produces the minimum force and is hence the preferred configuration. Configuration 1 with 4 cables is ‘preferable’ among all configurations despite requiring 4 motors due to the following:

1) The motors required are of less power and similar specification unlike other demanding higher power motors with different specifications.
2) The forces exerted by the device on the user’s limb are minimal.
3) The cable tension peak remains minimal throughout the gait cycle (except at the end of the gait cycle which is similar in all configurations).
4) The tracking performance is similar to other configurations.

#### Generating the Metrics Table

Based on the discussed metrics, a table was developed to help identify the most ‘suitable’ configuration for exoskeleton development. A suitable weight is assigned to each metric and can be adjusted depending on the priority and/or constraints given in terms of performance, model requirements, etc. The score assigned in the table is based on the performances and requirements as observed above. The minimum error during tracking, minimum requirements, and higher performances are given maximum scores and vice versa.

Configuration 1 has the highest score (Table 3) and is considered the most “suitable” configuration in this study. The addition of a spring improves the joint angular velocity tracking in configuration 3, however, the overall score is less than the 3-cable configuration without a spring (Configuration 2).

**TABLE 3:** General metric table for decision making.

Metric names		Weights	Score			
			Config 1	Config 2	Config 3	Config 4
Angle Error		20	20	18	20	16
Angular Velocity Error		20	16	18	20	16
Ankle Position Error		10	8	10	9	8
Cable Tension		5	5	3	3	3
Motor Torque *vs*. Speed (T-S) Curve		5	5	3.5	2	4
Area under T-S curve		10	8	10	7	9
Power Demand		10	7	9	10	8
Component Forces	Shear	10	10	7	7	8
	Compressive	10	10	7	7	8
Total Score		100	89	85.5	85	80

The metric-based identification methodology can be implemented to find the best cable routing of a configuration by creating multiple cases with different cable routing parameters (cuffs parameters). An optimization problem with the combination of the metrics as the objective function can be solved to find the optimal cuff parameters.

#### Identifying Motor Specification or Selecting Configuration Based on Available Motor

The actuator torque versus speed curve (T-S diagram) plotted in Figure 9 can be utilized to identify the motor specifications for the selected configuration. For an instant, the motors required to track the trajectory in configuration 1 are required to have the capability of generating 5 Nm torque at 300 RPM. Similarly, the plot can also be utilized in a reverse manner to select the configuration where a predefined motor specification (for example a certain torque at a certain speed), is provided. Assuming a motor capable of providing 5 Nm torque at 420 RPM is provided as a constraint, Figure 9 can serve as a baseline to find the suitable configuration based on the provided motor/actuator specifications (configurations 1 and 4).

## Conclusion

Cable driven rehabilitation devices are promising and capable of reshaping the field of robotic rehabilitation due to multiple advantages over traditional link-based devices. These include lightweight, exertion of negligible inertia and inertial vibration on the impaired limb, remote actuation capability, flexible routing and reconfiguration options, and flexibility in terms of exact joint alignment. Despite these benefits, the optimal number of cables and cable configurations to achieve the best performance remains elusive.

We have developed a generalized methodology/framework to model and assess cable-driven exoskeletons based on various metrics. The model assumes the lower limb as a two link-pendulum with the foot attached perpendicularly to the shank. The voluntary contribution of the user limb is assumed to be the only passive elastic joint moment. The proposed framework can be used to study different cable configurations, identify the suitable one and its associated optimal routing parameters, and estimate the required motor specifications to meet the tracking performance under specified constraints (such as pre-specified actuator T-S specifications). For proof of concept, four different configurations of a conceptual CDRD model that can apply the joint torque in an antagonistic arrangement were studied as potential models to develop a stroke rehabilitation lower limb exoskeleton. A spring was incorporated into one of the models to study the possibility of alteration in the model’s energy requirements along with other performance and requirements. A decision metric was developed based on the performance, requirements, and resulting forces applied to the joints to serve as a qualitative tool for the selection of a “suitable” configuration. Simulation of the conceptual configurations revealed that the 4-cable configuration is “suitable” for a lower limb exoskeleton development, as it generates minimum cable tension distribution, requires smaller individual motor power requirements, and produces a lower component force on the limb joints, as compared to the other configurations. The incorporation of spring in configuration 3 tended to increase the total power requirement of the CDRD (however at the lowest peak power demand pattern) but improved the overall tracking performance.

Some of the limitations of the current modelling approach include considering only the sagittal plane motion, assuming the user’s limb contribution as passive elastic joint moment only and assisting the user during the swing phase of the gait cycle. Moreover, the proposed framework is open to parameter adjustments including cuffs, cable tensions, springs, routing, etc., to meet the specified design objectives. Future work includes the extension of the current work to accommodate knee and hip abduction/adduction and cable routings in the frontal plane. Furthermore, the actual user limb contribution can be incorporated into the model instead of the passive elastic joint moment only if the impaired gait kinematic/kinetic trajectory is known.

## Data Availability

The original contributions presented in the study are included in the article/Supplementary Material, further inquiries can be directed to the corresponding author.
