# Supplementary figures and images for: A Framework for Determining the Performance and Requirements of Cable-Driven Mobile Lower Limb Rehabilitation Exoskeletons
Source: Front Bioeng Biotechnol. 2022 Jun 20;10:920462. doi: 10.3389/fbioe.2022.920462 (PMC9251017; doi:10.3389/fbioe.2022.920462)

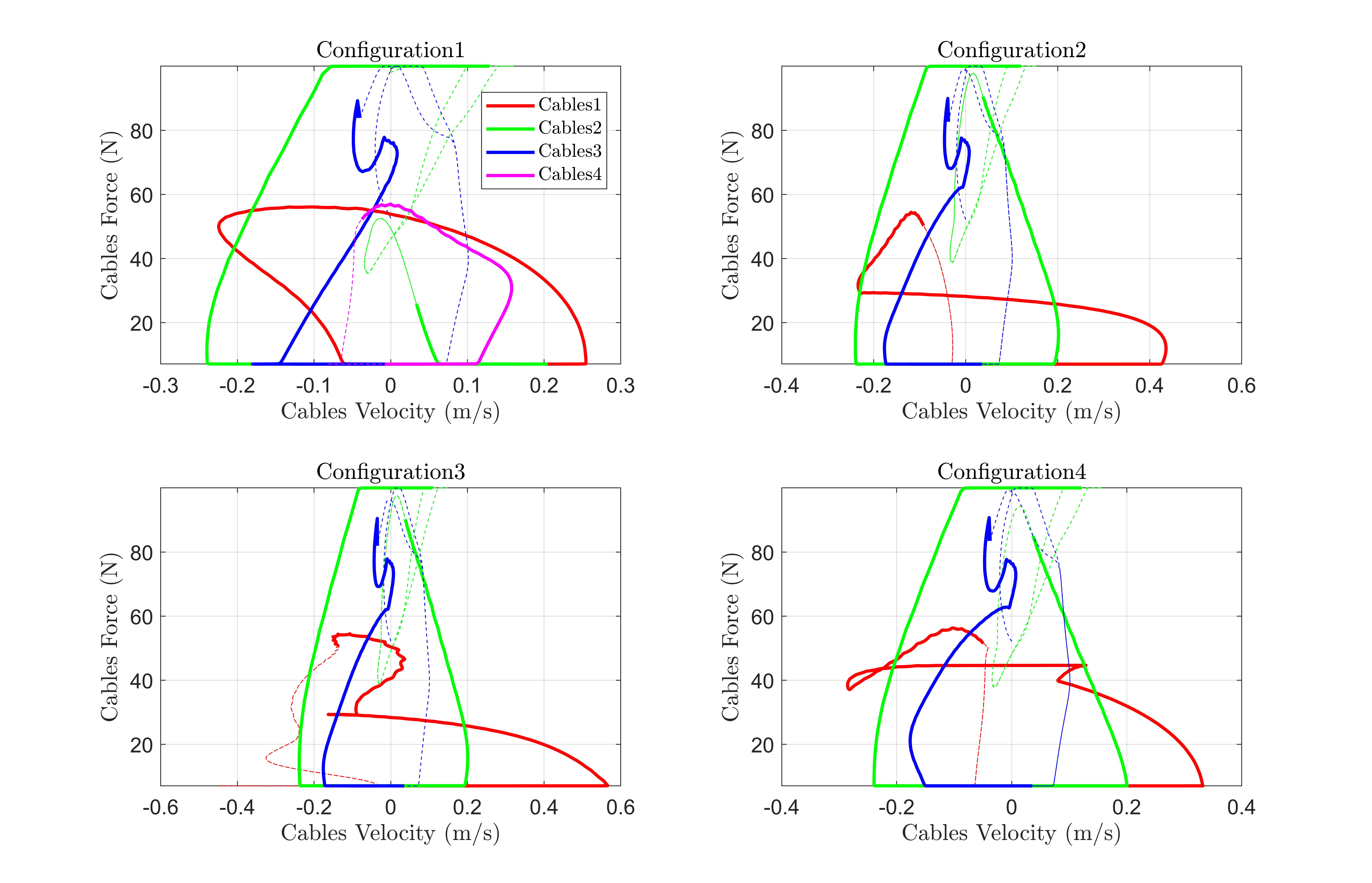

Supplement: Supplementary file 1 [file Image3.JPEG]

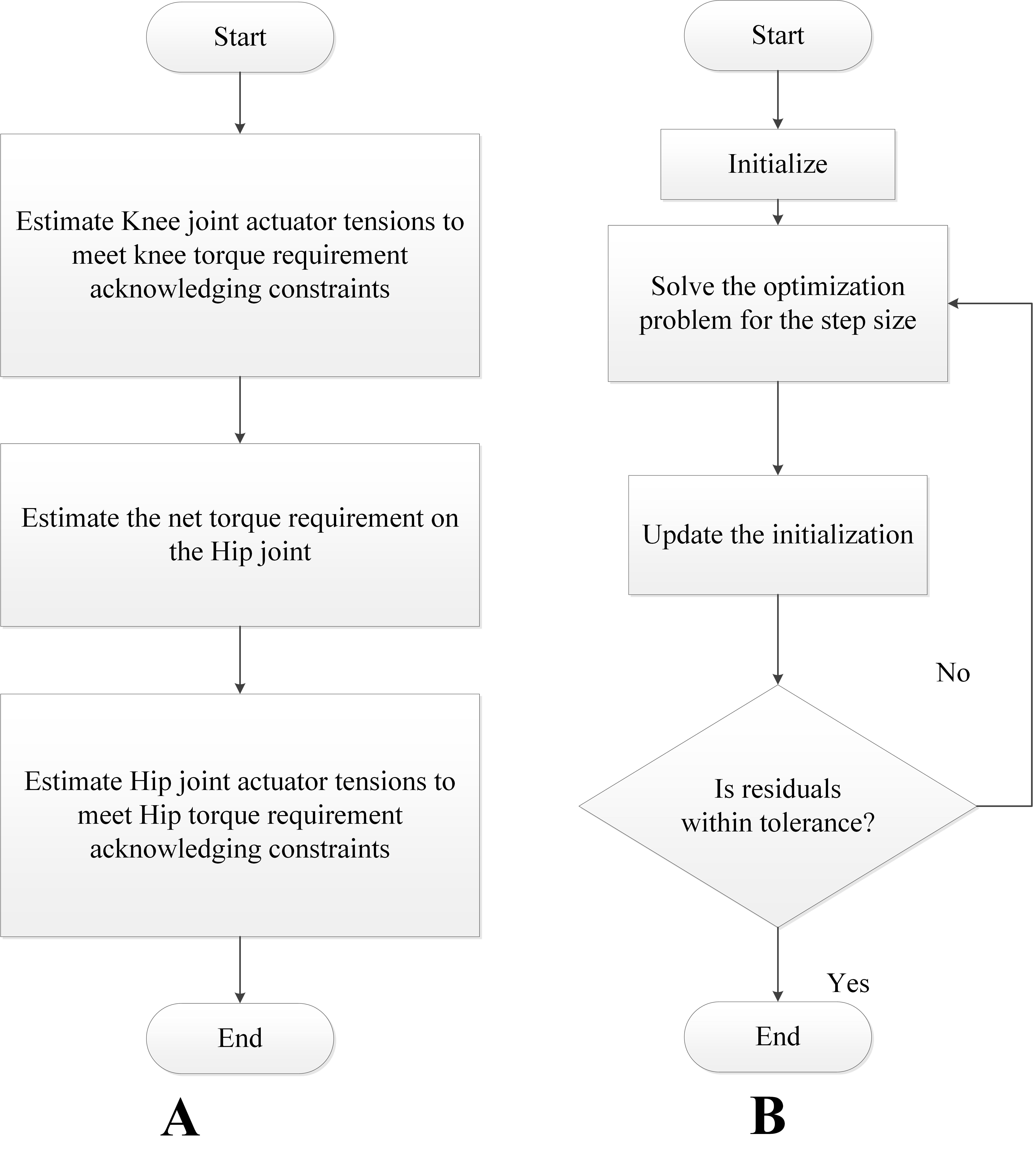

Supplement: Supplementary file 2 [file Image1.JPEG]

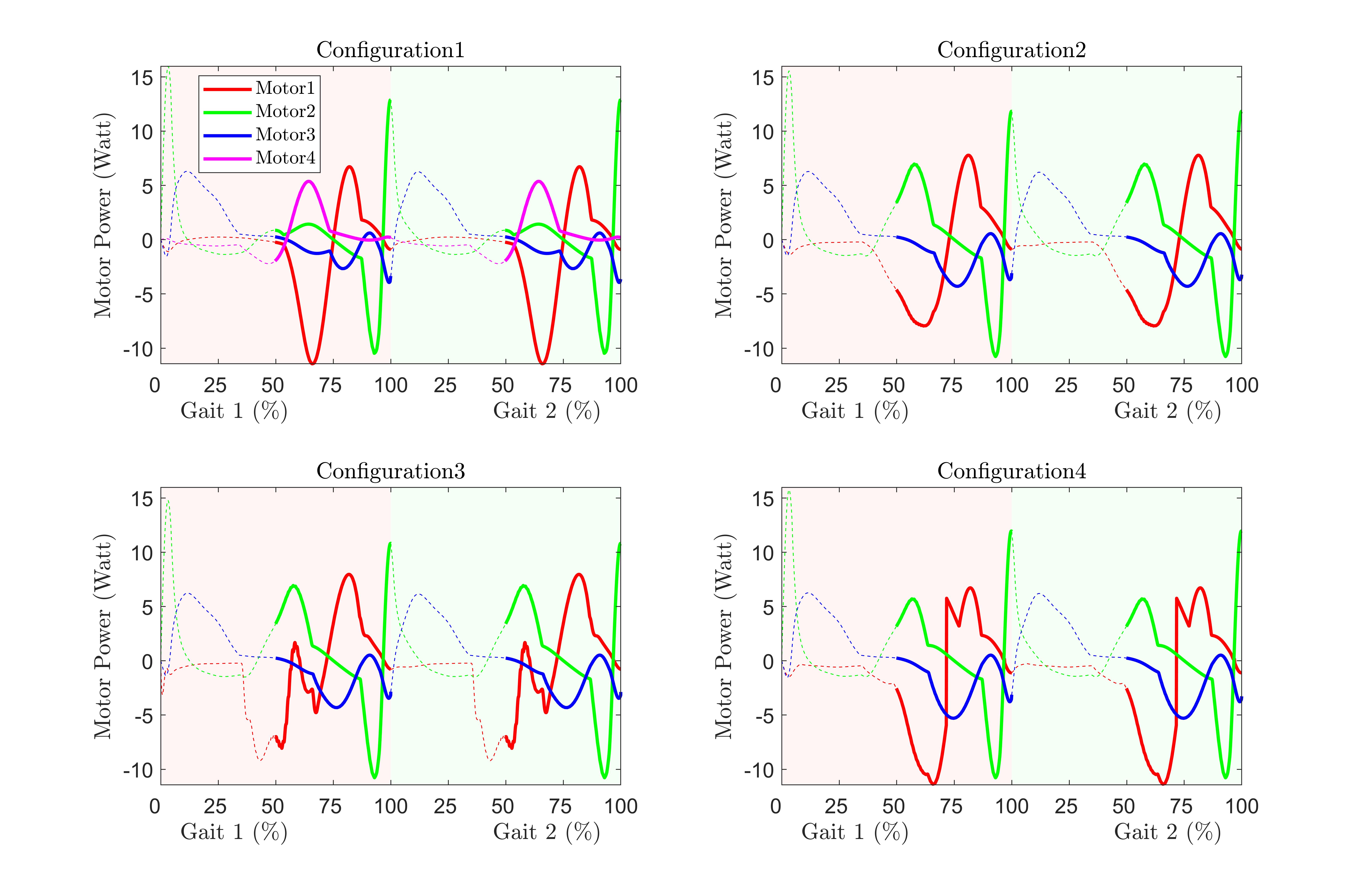

Supplement: Supplementary file 3 [file Image4.JPEG]

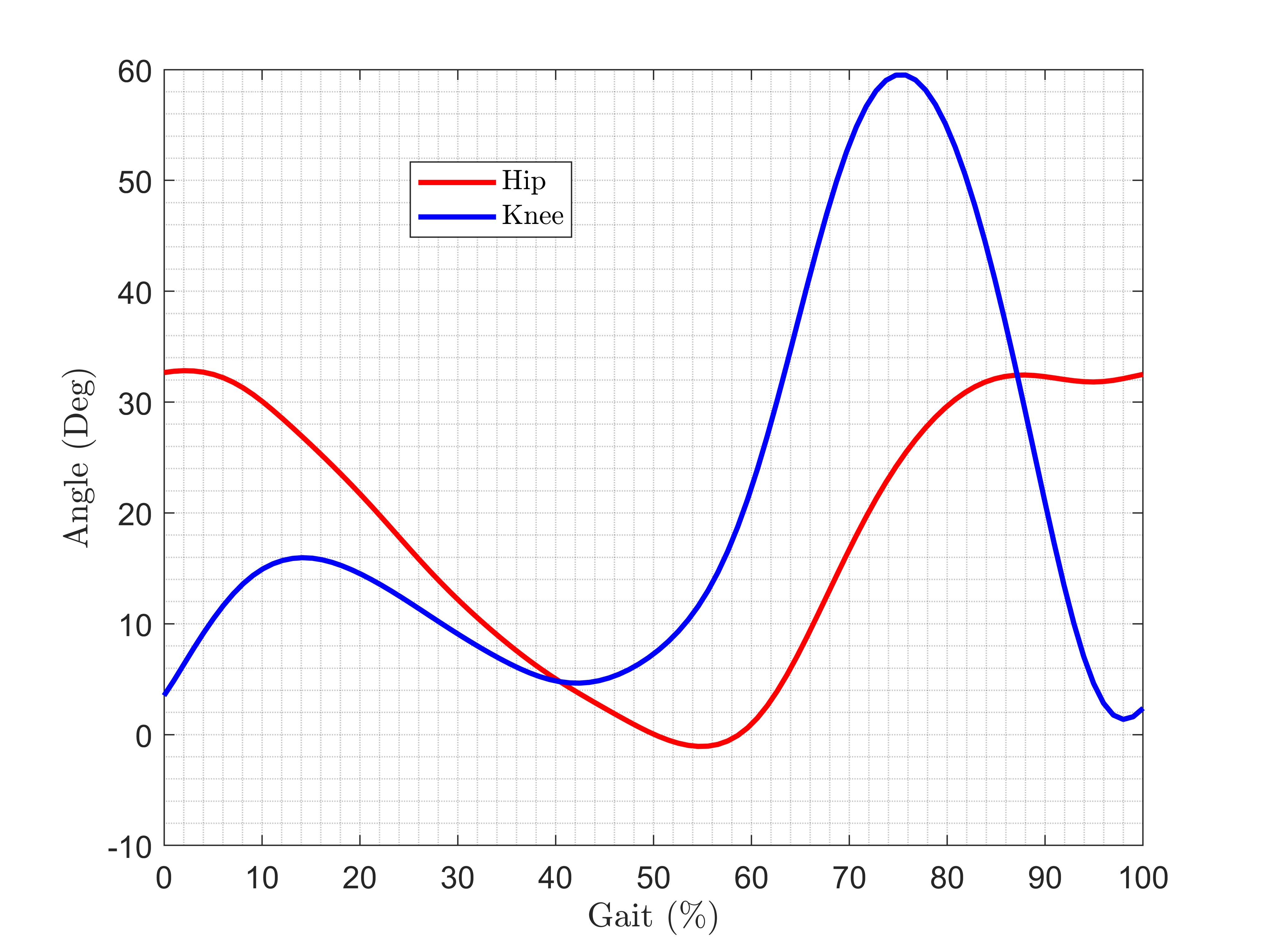

Supplement: Supplementary file 4 [file Image2.JPEG]
